# Competition Is a Strong Driving Factor in Wetlands, Peaking during Drying Out Periods

**DOI:** 10.1371/journal.pone.0130152

**Published:** 2015-06-15

**Authors:** Amandine Merlin, Anne Bonis, Christian F. Damgaard, François Mesléard

**Affiliations:** 1 UMR 6553 ECOBIO: Ecosystems, Biodiversity, Evolution, CNRS-University of Rennes 1, OSUR, Rennes, France; 2 Centre de recherche de la Tour du Valat, Arles, France; 3 Aarhus University, Department of Bioscience—Plant and Insect Ecology, Silkeborg, Denmark; 4 Institut Méditerranéen de Biodiversité et d’Ecologie (IMBE), IUT Université d'Avignon et des Pays de Vaucluse, UMR CNRS 7263 IRD 237 Aix Marseille Université, France; University of Sydney, AUSTRALIA

## Abstract

The aim of the study is to investigate the relative importance of plant-plant interactions with regard to flooding and drought effect on perennial plant performances in wetlands. Flooding is expected to be the major driver and, accordingly, the importance of drought is hardly if ever taken into account. Focusing on five widespread species, the growth, the survival and the competitive ability of plants were monitored on permanent plots spread along two elevation gradients. Flooding duration and drought intensity were found to vary substantially along the ~ 0.5 meter range elevation gradient. Flooding and drought alternate over the hydrological year and the pin-point surveys were thus conducted over the course of one year. The data were modeled taking into account survival, recruitment and competitive growth throughout flooding and drying out periods. Flooding and drought both directly impacted the plant performances and their competitive effect, with the effect of drought being much more general among species and of higher magnitude than flooding. The importance of competition was found to be high for all species, particularly during the drying out period. It varied more along the flooding gradient than along the drought gradient. The higher flooding tolerance shown by the studied species compared to drought may be related to species specific growth timing together with efficient response traits. These results offer new insights into the filters operating over the species pools. This suggests that the drying out period and drought conditions may be even more important for species’ relative success and the importance of competition than the flooding pattern. The general applicability of this result, obtained in mild Atlantic climate and fertile wetlands, remains to be studied.

## Introduction

The functioning of wetlands has been widely investigated, taking into account the alternation of flooding and drying out, e.g. [1, 2, 3]. By contrast, recent papers investigating plant performance and biotic interactions in wetlands have only considered the flooding pattern [4, 5, 6, 7, 8, 9]. Some others papers have considered the complete hydrological cycle, but focused only on the final plant performance without distinguishing the flood and dry out periods [10]. The suggestion by [11] of quantifying plant stress in relation to flooding (anoxia stress) and drying out (drought stress) thus constitutes a notable exception. It was accordingly demonstrated [12, 13] that these stress gradients were pertinent for the definition of the hydrological niche of wetlands species

Assessment of the importance of biotic interactions in wetlands must thus be conducted while taking into account the impact of flooding and drought for perennial plants. According to the Stress-Gradient Hypothesis [14], stress and disturbance modulate the nature and magnitude of biotic interactions: facilitation is expected in stressful habitats, whereas competition is expected to peak where stress or disturbances are milder. Several studies generally support this hypothesis (see meta-analysis by [15]), while species-specific and specific stress-type response patterns [16, 17, 18] rule out its use as a general pattern.

A wide range of parameters have been proposed for investigating plant-plant interaction strength. Here, we consider i) species’ competitive effect, i.e. its ability to deplete resources for others [19], and ii) competition importance, i.e. the reduction of the fitness of a plant species by the presence of competitors in relation to any other factor that influence plant fitness [20]. Competition importance offers a basis for appreciating the relative importance of competition against any other factors effect (here abiotic stress) and was for this reason chosen preferentially to the competition intensity which focuses on the effect of neighbors only [20]. Competition importance was previously found to increase along with productivity [21]. In flooded grasslands, [5] found that competition importance was peaking at one side of the flooding gradient or the other, depending on the ecology of the species. A similar result was shown along salinity gradients [22, 23, 24]. Such contrasts in the responses to environmental gradients may be related to species-specific response and to non-univocal effect of the environmental gradient, which may act as stress or not depending on the level considered and its timing.

To our knowledge, no work has yet documented how plant performances, plant competitive effect and competition importance vary with aeration stress, related to flooding, and with drought stress, occurring during the drying out of the wetlands.

Competition between species has been repeatedly reported to impact plant survival, recruitment and growth [25, 26]. These life-cycle parameters are thus appropriate to measure the role and importance of competition [27, 28], notably in natural communities [29] and to dissect out the various processes influencing the dynamics of populations (e.g. [28]). A model quantifying the importance of competition along plant density and environmental gradients has been developed by [30] and used to explain the results of the modeled competitive interactions.

In this study, this model framework was used to investigate plant performances, plant competitive effect and competition importance on the basis of field-based vegetation surveys in dense wetland vegetation. They were investigated along elevation gradients, 0.5 m wide on average, which drives an aeration stress gradient-during the flooding period- and a drought intensity gradient –during the drying out period- as shown by [11].

Plant surveys were conducted on permanent plots, spread out along the elevation gradient. Each permanent plot was characterized regarding the flooding and drought magnitude following [11].

Our aim was to study the effect of the flooding gradient and of the drought gradient, i) on the performances and on the competitive effect of five target species presenting different occurrence patterns *in situ* regarding elevation, ii) on the importance of competition for the success of the target species. We also aimed to compare the magnitude of the competition importance along the flooding gradient and along the drought gradient.

## Methods

### Study site and characterization of the environmental gradients

This study was conducted on two grazed wet grasslands situated in the Marais poitevin, on the French Atlantic coast (46°28’N; 1°13’W). We acknowledge the cooperation of the Mairie de Magnils Reigniers for granting permission to work in the common and of the Parc Naturel Régional du Marais poitevin and the Etablissement Public du Marais poitevin for their support in maintaining the experimental settings. The climate is of mild Atlantic type, with an excess of precipitation over evapotranspiration in winter of 220 mm on average and a water deficit in summer of about 300–350 mm [31]. These wet grasslands are fertile [32] and show high productivity for temperate permanent grasslands, up to 800 g dry matter/m² [33].

Annual cycles of flooding are typical of wet grasslands in temperate zones, with flooding occurring in autumn according to the amount of rainfall, and drying out of the marshlands generally occurs in June, but may be as early as April some years, depending on the rainfall pattern [7]. The soil elevation varies within these wetlands, ranging between 0.36 and 0.54 m. The flooding duration and the soil drought intensity vary depending of the position along the elevation gradient, respectively during the flooding and the drying out period [7, 34]. In accordance with [11], we quantified the soil aeration-shortage and drought, respectively, in relation to soil flooding and drying out. Following [34], the flooding duration was characterized as the Sum Exceedence Value above -0.19 m (thereafter aeration SEV, SEVa) and the drought intensity as the Sum Exceedence Value below the threshold of -0.42 m (thereafter drying SEV, SEVd) [11] (see S1 Appendix). The SEV parameters were derived from the water-table depth level, monitored at hourly intervals all year round using level logger sensors. SEVa summarizes the aeration-stress throughout the flooding period and SEVd summarizes the drought conditions throughout the drying out period.

In the two grasslands studied, the SEVa was found to vary from 0.3 and 12.8 cm.day^-1^, a similar range to that found in British alluvial grasslands [12]. The SEVd varied between 28.3 and 45.4 cm.day^-1^, showing locally drier conditions than in the British wetlands [12], probably due to the highly clay-rich soil together with large rainfall deficit during the summer.

### Vegetation survey

#### Studied species

We focused on five clonal perennial species with contrasted *in situ* distribution along the elevation gradient [7]. *Cynosurus cristatus* and *Lolium perenne* are mesophilous species and occur more frequently at higher elevations. *Juncus gerardi*, a meso-hygrophilous species, is found at intermediate elevations and *Glyceria fluitans*, a hygrophilous species, is mainly recorded at lower elevations. *Agrostis stolonifera* is distributed over the whole the elevation gradient.

#### Plant abundance measurements

Seventy permanent plots, 25 x 25 cm large, were placed every 20 cm along two elevation transects used as replicates (35 plots per sequence). On each plot, vegetation relevés were performed along both diagonals using the pinpoint method: a record was made every 4 cm for a total of 17 points per plot.

Three readings were made: on the 23–29 October, 2008, on the 3–12 June, 2009 and on the 19–20 October, 2009. The studied wet grasslands are usually grazed each year from April to November. Grazing exclusion was necessary in order to survey plant performances along the flooding and drought gradients without any confusing effect due to livestock grazing which may be selective and heterogeneous [33]. Fences were therefore established for one year in the two grasslands, a period short enough to avoid any significant change in the vegetation species composition and relative abundance.

Plant surveys consisted in measuring the vertical density and the cover of each species in each plot. The vertical density of a species i, Yi, represents the number of times that species touches each of the 17 pins in the pinpoint frame. The cover of species i, Xi, is the number of times that species is present at each point of the pinpoint frame. The vertical density is assumed to be a function of the cover of species during the growing season [35] as the change in vertical density is assimilated to the biomass growth within the growing season. The use of pin point field surveys and modeling following [30] constitutes a field approach to plant performances.

### Competition model

During the flooding period, i.e. from October 2008 to June 2009, the cover of species is assumed to be a function of the vertical density of species at the end of the previous growing season. Cover can be assimilated to the survival and recruitment abilities of species in response to the environment. In this way, the processes controlling the translation of biomass into cover over the flooding period and the effect of the environment on the processes controlling the biomass growth over the drying out period can be studied. More specifically, it is assumed that the vertical density of species *i* at time *t*2 is an increasing function of cover of species *i*, function of the cover of species *j* and *k* at time *t*1, and function of the environmental gradient *z*_*r*_. Competitive growth of species i was modeled as:

$$Y_{\left(i,t2,y,r\right)}=a_{i}\left(z_{r}\right)X_{\left(i,t1,y,r\right)}^{b_{i}}\text{exp}\left[\left(-c_{j}\left(z_{r}\right)X_{\left(j,t1,y,r\right)}^{d_{ij}}\right)\left(-c_{k}\left(z_{r}\right)X_{\left(k,t1,y,r\right)}^{d_{ik}}\right)\right]+\varepsilon_{\left(S1,i,y,r\right)}$$ (1),

with r the pin-point frame; the residual process variation during the flooding period of species i across different years and pin-point frames. *a*_*i*_(*Z*) corresponds to the growth of species i directly affected by the relation between the cover and the vertical density of the species; *c*_*j*_(*Z*) correspond to competitive effects of species *j* affecting growth of species *i*. The model assumes that the competitive effect of species *i* on species *j* is equal to the competitive effect of species *i* on species *k*. While oversimplified, we consider this assumption to be acceptable, as the main purpose of this work is to untangle the competition versus environmental effect on species performances. The parameter functions *a*_*i*_(*Z*) and *c*_*j*_(*Z*) are functions of the environmental gradient as linear functions, *a*_i,0_ + *a*_i,1_ Z and *c*_j,0_ + *c*_j,1_ Z, respectively. This means that *a*_i,1_ and *c*_j,1_ measured the effect of the environmental gradient (*z*) on growth and the competitive effect of species *j*, respectively. In this study, *z* is the value of aeration SEV (when considering flooding effect) and is the value of drying SEV (when considering drought effect).

Flooding occurred at the end of autumn and ended in spring of the following year: recruitment and survival abilities of species are integrative of the fitness of the species during this period [35]. Over the period of flooding, it is assumed that the cover of species *i* at year *y*+1 (June, 2009, here) is an increasing function of the vertical density of species at year *y* (October, 2008) at the end of the growing season), and a decreasing function of the vertical density of species *j* and *k* at year *y* and together a function of the environmental gradient *z*. Survival and recruitment of species i at year *y*+1 was modeled as:

$$X_{i,t1,y+1,r}=a_{i}\left(z_{r}\right)Y_{i,\;t2,y,r}^{\;b_{i}}.\;\text{exp}\left[\left(-c_{j}(z_{r})Y_{j,\;t2,y,r}^{\;d_{ij}}\right)\cdot \left(-c_{k}(z_{r})Y_{k,\;t2,y,r}^{d_{ik}}\right)\right]+\varepsilon_{S1,i,y,r}$$ (2)

with the residual process variation from one season to the next of species i among years and pin-point frames.

The competition model has previously been used to successfully model the effect of herbicide and nitrogen on survival, recruitment, and competitive growth of two perennial grass species from similar time series pin-point data [36, 37]. In this study, it will monitor the result of actual interactions between plants at different life-history stages and after the long term run of the assemblages.

As this model is a ‘three species model’, the different species surveyed were aggregated into three groups: the first group corresponded to *Agrostis stolonifera*, the second group corresponded to, alternatively, *Lolium perenne* (Lp), *Cynosurus cristatus* (Cc), *Juncus gerardi* (Jg), *Glyceria fluitans* (Gf), and a third group (“other aggregated species” in S1 Table) comprised the rest of the higher plant species recorded on the plot.

### Parameter estimation

Model parameters (a, b, c and d) were estimated using the Bayesian method: the joint posterior distribution of model parameters was calculated using the MCMC method (Metropolis-Hastings algorithm with 120000 iterations) and a multivariate distribution. The sampled chains of all parameters were inspected to check the properties of the sampling procedure. The effect of the environmental conditions on species performances (parameter a1) and on species competitive effect (parameter c1) were assessed by the 2.5%, 50% and 97.5% percentiles provided from the marginal posterior distribution of the parameters. For a_1_ value significantly larger than zero, species growth increases with SEV. Likewise, if c_1_ is larger than zero, then the competitive effect of the species increases with SEV.

Growth was compared among species pairs modeled together: when their 95% credible intervals for a_1_ do not overlap, then it significantly differs from one to another.

### Assessment of the importance of competition

From Eqs (1) and (2), the importance of competition was quantified using the competition models (1) and (2) and the measured cover and vertical densities (i) during the flooding period, i.e. comparing surveys in October, 2008, and in June, 2009, (3) and (ii) during the summer period, comparing data in June and October, 2009 (4), following the proposition of [30]:

$$\frac{\left|\partial_{Y_{j}}\;X_{i}\;\left(Y_{j},z\right)\right|}{\left|\partial_{Y_{j}}\;X_{i}\;\left(Y_{j},z\right)\right|+\left|\partial_{z}\;X_{i}\;\left(Y_{j},z\right)\right|}$$ (3)

$$\frac{\left|\partial_{X_{j}}\;Y_{i}\;\left(X_{j},z\right)\right|}{\left|\partial_{X_{j}}\;Y_{i}\;\left(X_{j},z\right)\right|+\left|\partial_{z}\;Y_{i}\;\left(X_{j},z\right)\right|}$$ (4)

Where $\left|\partial_{X_{j}}\;Y_{i}\;\left(X_{j},z\right)\right|$ and $\left|\partial_{Y_{j}}\;X_{i}\;\left(Y_{j},z\right)\right|$ represent the changes in the measured ecological success of the species *i*, as modeled by Eqs (1) and (2) by changing the levels of the environmental conditions *z*.

## Results

### How do environmental conditions impact species’ performances?

**Fig 1:**
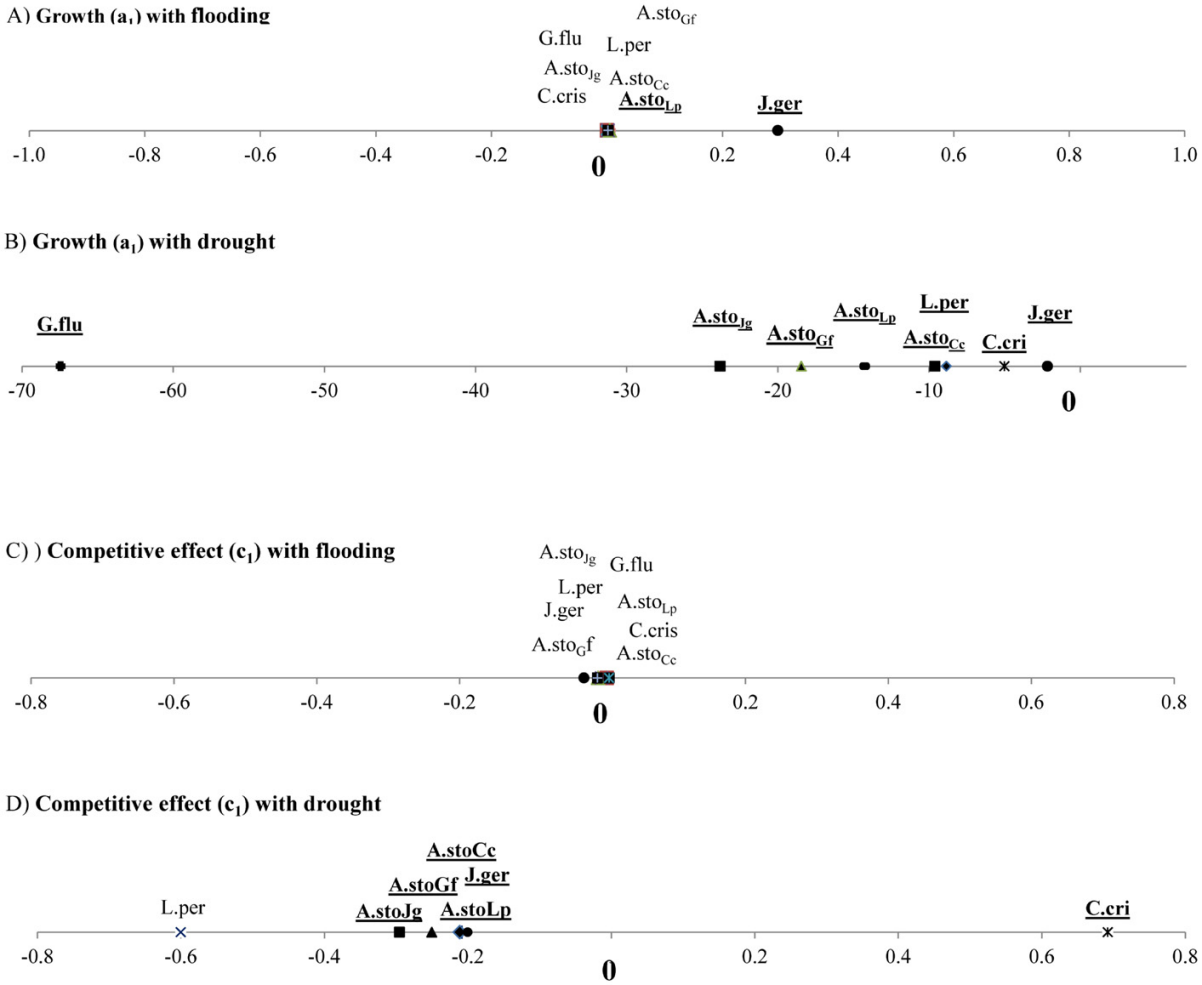
Median of a_1_ estimating specific plant growth and c_1_, estimating species competitive effect, along flooding (A, C) and drought gradients (B, D). A positive value indicates that flooding (drought) promotes growth or competitive effect while negative value indicates a detrimental effect of flooding (drought) on growth or competitive effect. Species name for which the trend is significant appear in bold and is underlined. For *Agrostis stolonifera*, growth and competitive effect are reported with the model run with four different species for which the initials appear in subscript (A.sto_Lp_, A.sto_Cc_, A.sto_Jg_, A.sto_Gf_).

The effect of the flooding duration and drought intensity on species’ performances (survival, recruitment and growth) were distinguished from the effect of biotic interaction and assessed by the percentiles of parameter a1 (Fig 1 and S1 Table). Species’ performances, regardless of the group considered, were found to be much higher during the drought period than during the flooding period.

The increase in flooding duration, approached by the aeration SEV, had a significantly positive effect on the survival and recruitment of *J*. *gerardi* and of *A*. *stolonifera*, only when modeled with *L*. *perenne* (A.sto_Lp_). It showed neither a significant effect on recruitment nor on survival for the other species, while this was particularly unexpected for *G*. *fluitans*. The increase in flooding duration had a significantly positive effect on the performances of the three ‘aggregated species’ groups out of the 4 (S1 Table).

Higher drought diminished the performances of all 5 studied species (*L*. *perenne*, *C*. *cristatus*, *J*. *gerardi*, *G*. *fluitans* and *A*. *stolonifera)* in all groups. Drought showed a more limited effect on *J*. *gerardi* compared with the other species. Increasing drought was found to be non-significant for the performance of 2 ‘aggregated species’ groups, negative for one and positive for one out of the 4 (Fig 1 and S1 Table).

### Effect of environmental conditions on species competitive effect

Increase in flooding duration showed no significant effect of the competitive effect of any of the 5 studied species (Fig 1 and S1 Table). It had only a positive effect on the competitive effect of in 3 out of the 4 “aggregated species” groups considered (S1 Table).

During the summer period, the competitive effect of *A*. *stolonifera* (in all groups), of *J*. *gerardi* and of all “aggregated species” groups was found to be diminished by increasing drought (Fig 1 and S1 Table). Only the competitive effect exerted by *C*. *cristatus* was improved by more intense drought conditions.

### Importance of competition

**Fig 2:**
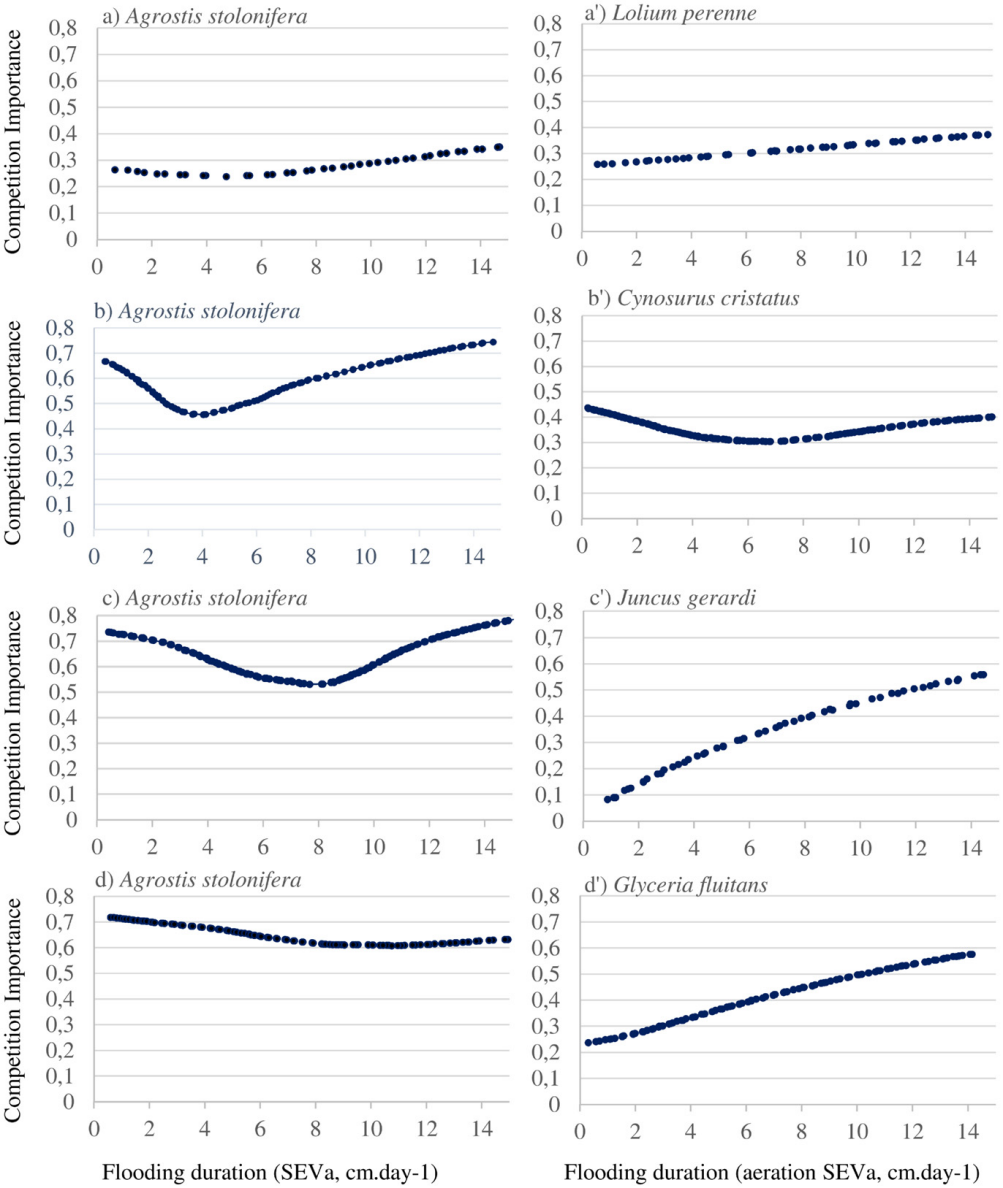
Importance of competition for each species along the flooding gradient with an initial cover of species, i, j and k: 1, 1, 0. The importance of competition represents the proportion of change in ecological success caused by competition relative to environmental conditions. The SEVa was calculated following [11] and expressed as cm.day-1. Fig 2 reports the results for *Agrostis stolonifera* modeled in pair with *Lolium perenne* (Fig 2a), *Cynosurus cristatus* (Fig 2b), *Juncus gerardi* (Fig 2c), *Glyceria fluitans* (Fig 2d). Results for *Lolium perenne* are reported in Fig 2a’, *for Cynosurus cristatus* in Fig 2b’, for *Juncus gerardi* in Fig 2c’ and for *Glyceria fluitans* in Fig 2d’.

The importance of competition represented the proportion of change in a species’ ecological success caused by competition relative to the impact of environmental conditions. Along the gradient of flooding duration, the competition importance showed two main patterns of variation, depending of the species. (i) For *L*. *perenne*, *J*. *gerardi*, *G*. *fluitans* and *A*. *stolonifera*-considered with *L*. *perenne-*, the importance of competition increased steadily with the flooding duration (Fig 2). For *J*. *gerardi* and *G*. *fluitans*, competition determined up to 60% of the plant performance, while this proportion remained lower than 40% for the other species. (ii) For *C*. *cristatus* and for *A*. *stolonifera -*when considered with *C*. *cristatus* and *J*. *gerardi* as species pair in the model-, the importance of competition varied within a slightly more limited range (30–40% and 50–80%, respectively) and was maximized at both ends of the flooding gradient (Fig 2).

**Fig 3:**
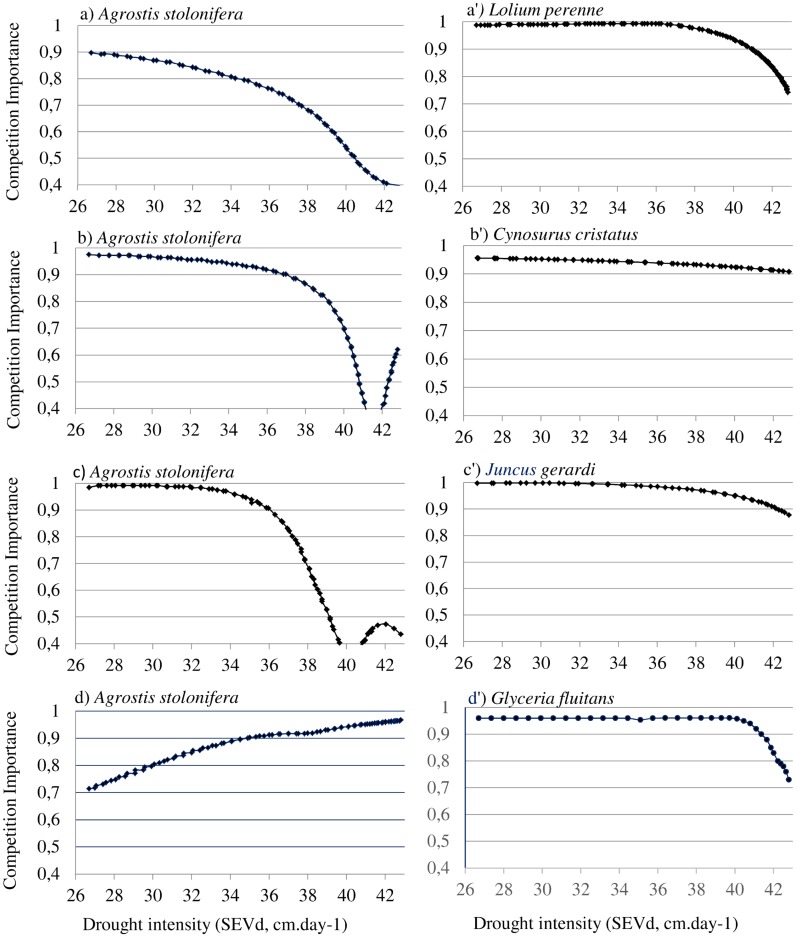
Importance of competition calculated for each group of species along the drought gradient, with an initial cover of species, i, j and k: 1, 1, 0. Competition importance represents the proportion of change in ecological success caused by competition relative to environmental conditions. The SEVd was calculated following [11] and expressed as cm.day-1. The Fig 3 (a, b, c, d) report the results for *Agrostis stolonifera* modeled in pair with respectively *Lolium perenne*, *Cynosurus cristatus*, *Juncus gerardi*, *Glyceria fluitans*. Results for *Lolium perenne* are reported in Fig 3a’, *for Cynosurus cristatus* in Fig 3b’, for *Juncus gerardi* in Fig 3c’ and for *Glyceria fluitans* in Fig 3d’.

During the drying out, the importance of competition was the highest for all studied species under milder water resource conditions (Fig 3). For *L*. *perenne*, *C*. *cristatus* and *G*. *fluitans*, the competition importance remained very high (> 90%) across a very wide range of drying SEV values and only decreased under very harsh-water limited- conditions, with drying SEV> 38 cm.day^-1^. For all three species, competition then almost entirely determined plant performances under the milder drought conditions. *A*. *stolonifera* showed the same pattern, but the competition importance showed a stronger diminution, being limited to 40% with the harsher drought conditions for this species, except when considered with *G*. *fluitans*.

## Discussion

This work has shown that in fertile wetlands, and with Atlantic mild climate, flooding is a poor direct driver for species performance and for species’ competitive effect. By contrast, drought impacts all five species’ performance by directly affecting their survival and growth. Drought also controls the competitive effect of 2 out of the 5 species studied. Both flooding and drought gradients drive competition importance for all species: species-specific patterns were found along the flooding gradient, whereas competition importance almost entirely determined the species’ performances and peaks as soon as drought stress was somehow relaxed. This result was obtained with grazing being temporary excluded: it may be expected that, with grazing, competition importance would have been milder as grazing diminished plant height, a good proxy for competition effect [38].

The importance of competition on species performances relative to flooding and drought appears high in wet grasslands, and flooding is thus not the predominant driver of the plant performance. This suggests that the general and only focus on the impact of flooding in wetland vegetation studies is not justified, as already shown by [39].

### The impact of gradients on plant performance

Flooding duration showed no significant impact for 3 out of the 5 perennial species studied, two mesophilous and one hygrophilous species. Those results fit well with [40], who also found that the elevation gradient did not significantly change biotic interferences. In agreement, [41] also found a limited and vanishing effect of flooding on plant performance and on competitive ability and hierarchy among species. This result may, however, be restricted to perennials which were found to be less responsive to flooding variation than annual plants [42].

It may in fact be questioned whether flooding constitutes a stress as defined by [43], as this study showed that no species’ performance was impacted by the flooding pattern. Results by [34] also supported the view that flooding is a poor filter of the local grassland’s species pools, as it impacted species richness to a very limited extent and did not significantly impact biomass production. This suggests that long lasting plant assemblages are, in the field, far less responsive to flooding pattern than what was reported from short term experiments [6, 44], or when focusing on biomass only [5]. It is thus strongly advised to consider long term assemblages and various stages of plant life-cycle over the whole ecological cycle to obtain a realistic picture of plants’ performances and biotic interactions across gradients in wet grasslands.

Two main explanations may be suggested for this limited impact of flooding regime:

1. Species that make up the species pool, either mesophilous, meso-hygrophilous or hygrophilous, are all able to cope with aeration stress (see [45]) as all the wet grasslands are subjected to water saturation in the soil for several weeks every year and over centuries. Flooding tolerance may indeed be actually shared by all species in the wetlands, at all elevations, due to previous filtering process of the species regional pool.
2. Growth timing pattern may both mitigate aeration stress and accentuate drought. Flooding mainly occurs when plants are passing through a period of vegetative rest, and the associated aeration stress may thus only have slight impact in contrast to drought, which occurs in more active periods. Accordingly, the growth performances modeled from the pin-point measurements (parameter a_1_) actually depict a much less active growth pattern from October to early June (flooding period) than from mid-June to October. The peak in N mineralization and thereafter N availability in June and July [46] also suggest that a major limiting factor for growth is alleviated in early summer.

Monitoring species and community growth patterns all-year-round will provide a basis for assessment of whether or not stress occurrence coincides with plant growth and whether this may be a plausible explanation for the limited effect of flooding on plant success.

The results obtained in this study also support the view that biotic interactions are key driven factors liable to explain plant communities’ and species’ relative abundance patterns along elevation gradients [31, 40], as also shown by [18] and [47]. The high plant biomass and density in the studied fertile wet grasslands [32] is probably a major promoter for the importance of biotic interactions in the assemblages.

### Species response to flooding

The two species that are significantly impacted by flooding duration, *J*. *gerardi* and *A*. *stolonifera*, actually benefitted from longer flooding with regard to their survival and recruitment. Under flooded conditions, *A*. *stolonifera* developed longer stems along which adventitious roots grew, which favored space occupancy and high re-growth ability [48, 49], together with good competitive effect [6]. Regarding *J*. *gerardi*, its high tolerance to flooding was first recognized by [50]. Its ability to store resources in rhizomes [51] may help cope with flooding and support spring growth, as shown for other species [52, 53].

The high tolerance to flooding shown by the three other species (i.e. *C*. *critatus*, *L*. *perenne* and *G*. *fluitans)* may be related to eco-physiological and morphological adaptations, as reported in abundant literature (see e.g. synthesis by [54, 55]) and may also imply clonal traits [56].

In contrast to flooding, soil drought was found to be effective in controlling species’ performance and species’ competitive effect for all five species. Their performances were found to increase with water availability and, consequently, their competitive effect (see [57, 58] for the relationship between plant biomass or size and competitive effect). The impact of drought was limited on *J*. *gerardi*, probably due to its early-growing pattern [59, 60].

Showing that reduced drought had a positive effect on plant performance is by no means unexpected. However, the magnitude of drought impact and its general occurrence suggests that it plays a much more important role in the vegetation pattern in wet grasslands than flooding, and this was unexpected. Vegetation patterns commonly reported along elevation gradients may accordingly be related to drought and water availability during the drying out period even more than to the flooding pattern.

At the community level, the weaker effect of flooding-related stress can be explained by the period of flooding: it occurs over the colder period, when the conditions are mainly unfavorable for plant perennial growth in temperate wetlands, and this may have thereafter limited plant-plant interactions.

### Plant-plant interactions and abiotic stress

It was predicted by [61] that abiotic stress limits species distribution at the harsher end of the gradient, while competition drives the vegetation pattern at the milder end. Referring to the Stress-Gradient Hypothesis [14, 61] stress and disturbance are thus expected to modulate the nature and magnitude of biotic interactions. Such a negative relationship between the importance of competition and environmental stress has been found by e.g. [5, 17, 21, 24] in tidal, wet, alpine and dry grasslands, respectively.

In the present work, a similar relationship was found in 7 out of the 8 situations studied with the competition importance reaching its maximum, determining up to 90% of individual performances, as soon as the drought level slightly decreased (i.e. SEVd <36). The detrimental effect of plant-plant interactions was thus minimized under the harsher drought conditions, as seen in dry grasslands [17]. During the flooding period, the importance of competition also varies with elevation, but was maximized at high anoxia stress levels for 6 out of 8 cases, then showing an inverse pattern with stress than during the drying out period.

To be realistic, vegetation patterns spread along elevation gradients must be investigated while considering both the increasing drought stress from low to high elevation during summer time and the increasing aeration stress from high to lower elevations during flooding. Predictions regarding the competition importance pattern along the elevation gradient will be opposed, depending on whether one or the other stress factor is considered. This argues in favor of standardized methods to characterize environmental gradients, as proposed for plant traits measurements [62], taking into account the complete hydrological cycle and plant critical stages.

World-wide investigations comparing plant-plant interactions between harsh and mild habitats yield conclusive results, although not unequivocally [63]. The different natures of the various stress and resources may explain the muddled picture frequently obtained in case studies. The studied wetlands vegetation presents a very different picture than that of alpine or saltmarsh communities where positive interactions were repeatedly found. As far as the importance of competition is concerned, this study, like [34], concluded that plant interaction plays a strong role in wet Atlantic grasslands. This study may shed new light on the increase of the Specific Leaf Area value at the community level found by [34] with flooding. This trait pattern was interpreted as a trait response to increasing aeration shortage. However, as the drying out was found to be the predominant growth period, increased SLA value at low elevation may also correspond to an increase in the growth ability of the community with decreasing drought.

## Conclusion

Both flooding and drought impact wetlands’ plant species survival and growth but drought effect was found to be much more effective for the perennial species studied, regardless of whether the plant species are mesophilous, meso-hygrophilous or hygrophilous. This picture also holds for the species competitive ability which was found to be significantly impacted by drought and not by flooding pattern.

The importance of competition has been found to be high both during the flooding and the drying out periods, while peaking during the drying out period. Under the milder drought conditions, it was found to determine over 90% of the plant performances, and again, the picture was similar for hygrophilous, meso-hygrophilous and mesophilous species. Competition importance did vary along the flooding and the drought gradients but the pattern is neither linear nor regular among species.

In contrast to the stress gradient hypothesis, competition was the dominant process throughout the stress gradient. This may be due to the homogeneity of the growth forms characterizing the plant communities studied, where 80% of the cover is made up of clonal *poaceae*. Rather than a negative test of the stress gradient hypothesis, we suggest that this poor growth form diversity may not be propitious to facilitation effect, due to the driving force of grazing on growth forms.

This study highlights the drying out period and drought which constitute the “hidden side” of the elevation gradient in wetlands. As a consequence, those responsible for managing these habitats for whatever biodiversity or agriculture purposes will have to take into account this novel information.

## Supporting Information

S1 Appendix Characterization of the aeration and drought stress magnitude. (DOC)

S1 Table Estimated effect of flooding and drought on species growth (*a*_1_) and species competitive effect (*c*_1_) summarized by the marginal posterior distribution of the parameters. If a_1_ is greater than zero, then growth of the species increased with SEV. Likewise, if c_1_ is greater than zero, then the competitive effect of the species increases with SEV. Parameters significantly deviated from zero appear in bold font. The species considered in pair in the model that are followed by similar letters showed non-significantly different growth.
(DOC)
