# Supplementary material for: Competition Is a Strong Driving Factor in Wetlands, Peaking during Drying Out Periods
Source: PLoS One. 2015 Jun 15;10(6):e0130152. doi: 10.1371/journal.pone.0130152 (PMC4468187; doi:10.1371/journal.pone.0130152)

**S1 Appendix. Characterization of the aeration and drought stress magnitude.**

**Characterization of the aeration and water shortage stress magnitude.**

A precise characterization of the waterlogging and drought constraints experienced by plants was first given in [11]. According to [11], the SEV for aeration (see below) drove mainly species success and community distribution, whereas drought did not. In this work, the aeration and drought stresses have been characterized by two indices which both consist in a Sum Exceedance Value (SEV). The SEV for aeration (SEVa) as the dryness SEV (SEVd) are expressed in cm.day^-1^ or in m.week^-1^. Each index consisted of determining the timing and the extent of the constraint by determining two thresholds from which stress occurs from March to September [11, 67, 68]. The aeration threshold corresponded to a water table depth of -0.191 m (upper line in the figure): when the water table level is above this threshold, waterlogging occurs. This corresponds to the grey area above -0.191 m in the figure.

The drought threshold corresponded to a water table of -0.42 m (lower line in the figure): when the water table level is under this threshold, drought occurs. This corresponds to the grey area below -0.42 m in the figure.

Water table level was monitored across time with automatic probes (Solinst LTC Levelogger Junior modell 3001) situated in dip wells placed *in situ* along the topographical gradient (4 dip wells par elevation gradient).

The SEV for aeration was calculated as the difference between the water table depth and the reference value (i.e. threshold) following this equation [69].

$$SEV= \sum_{t=1}^{T} EW$$

where

$$EW_{t}=RV-WT_{t} WT_{t}<RV$$

$$EW_{t}=0$$

$$WT_{t}>RV$$

WT_t_ is the water-table depth at time t from March to September and RV the reference water-table depth above which the plants are expected to be aeration stressed. The reference water-table depth is a threshold calculated from a soil moisture release curve as the depth that gives 10% air-filled porosity, inducing an insufficient oxygen diffusion to supply the respiratory demands of roots during growth period (11, 69). It corresponds to - 0.191 m.The dryness SEV was calculated using the same formula but considering water table below -0.42 m as threshold


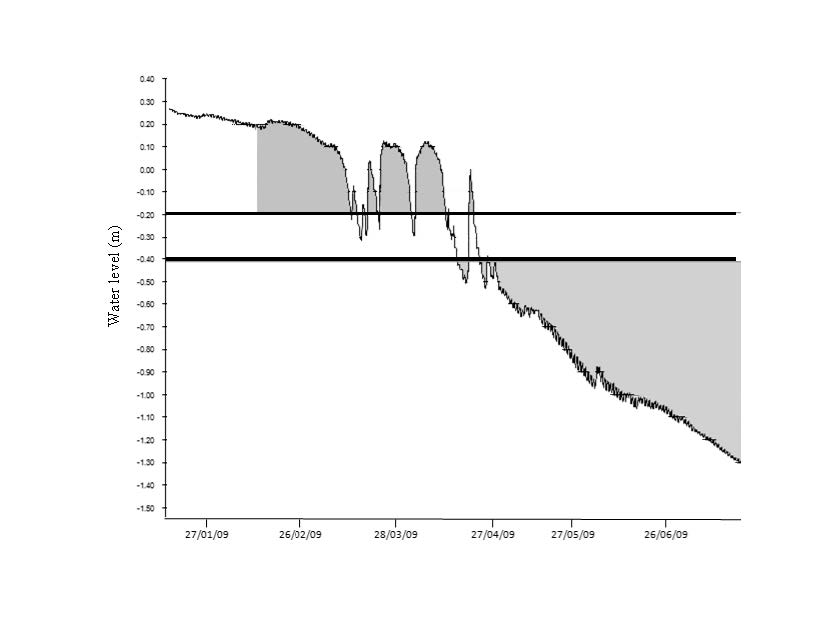

Supplement: S1 Appendix — (DOC) [file pone.0130152.s001.doc]
