# Supplementary material for: Competition Is a Strong Driving Factor in Wetlands, Peaking during Drying Out Periods
Source: PLoS One. 2015 Jun 15;10(6):e0130152. doi: 10.1371/journal.pone.0130152 (PMC4468187; doi:10.1371/journal.pone.0130152)
Supplement: S1 Table — If a1 is greater than zero, then growth of the species increased with SEV. Likewise, if c1 is greater than zero, then the competitive effect of the species increases with SEV. Parameters significantly deviated from zero appear in bold font. The species considered in pair in the model that are followed by similar letters showed non-significantly different growth. (DOC) [file pone.0130152.s002.doc]

**S1 Table. Estimated effect of flooding and drought on species growth (*a*_1_) and species competitive effect (*c*_1_) summarized by the marginal posterior distribution of the parameters**.

|  | | **a_1_** | | | **c_1_** | | | | |
| --- | --- | --- | --- | --- | --- | --- | --- | --- | --- |
| **Credible interval** | | 2.5% | 50% | 97.5% | 2.5% | | 50% | 97.5% | |
| **Flooding** | *A. stolonifera* (a) | **0.0001** | **0.003** | **0.009** | -0.006 | 0.001 | | | 0.007 |
|  | *L.perenne* (a) | -0.004 | 0.002 | 0.018 | -0.023 | -0.001 | | | 0.013 |
|  | Other aggregated species | -0.0006 | 0.030 | 0.103 | **0.003** | **0.018** | | | **0.034** |
| **Flooding** | *A. stolonifera* (a) | -0.004 | 0.0001 | 0.003 | -0.006 | 0.005 | | | 0.022 |
|  | *C. cristatus* (a) | -0.005 | -0.002 | 0.004 | -0.001 | 0.009 | | | 0.029 |
|  | Other aggregated species | **0.560** | **0.630** | **0.723** | -0.004 | 0.005 | | | 0.014 |
| **Flooding** | *A. stolonifera* (a) | -0.003 | 0.0005 | 0.005 | -0.003 | 0.006 | | | 0.016 |
|  | *J. gerardi* (b) | **0.194** | **0.296** | **0.446** | -0.057 | -0.026 | | | 0.008 |
|  | Other aggregated species | **0.935** | **1.109** | **1.412** | **0.004** | **0.017** | | | **0.030** |
| **Flooding** | *A. stolonifera* (a) | -0.002 | 0.0005 | 0.005 | -0.018 | -0.006 | | | 0.0002 |
|  | *G. fluitans* (a) | -0.001 | 0.002 | 0.004 | -0.037 | -0.007 | | | 0.006 |
|  | Other aggregated species | **0.906** | **1.047** | **1.149** | **0.002** | **0.581** | | | **0.973** |
| **Drought** | *A. stolonifera* (a) | **10.115** | **14.415** | **31.355** | **0.167** | **0.208** | | | **0.262** |
|  | *L.perenne* (a) | **4.453** | **9.610** | **17.900** | -0.355 | -0.060 | | | 0.081 |
|  | Other aggregated species | -4.219 | -2.340 | 1.0152 | **0.048** | **0.074** | | | **0.115** |
| **Drought** | *A. stolonifera* (a) | **6.819** | **8.855** | **11.196** | **0.190** | **0.211** | | | **0.223** |
|  | *C. cristatus* (b) | **4.971** | **5.022** | **5.694** | **-0.940** | **-0.692** | | | **-0.497** |
|  | Other aggregated species | **-5.760** | **-3.886** | **-2.012** | **0.063** | **0.067** | | | **0.088** |
| **Drought** | *A. stolonifera* (a) | **20.337** | **23.812** | **27.136** | **0.278** | **0.295** | | | **0.316** |
|  | *J. gerardi* (b) | **2.017** | **2.158** | **2.528** | **0.130** | **0.200** | | | **0.274** |
|  | Other aggregated species | **17.176** | **22.662** | **31.033** | **0.073** | **0.090** | | | **0.109** |
| **Drought** | *A. stolonifera* (a) | **10.916** | **18.438** | **31.817** | **0.225** | **0.250** | | | **0.283** |
|  | *G. fluitans* (b) | **47.157** | **67.449** | **100.842** | -0.144 | 0.208 | | | 0.638 |
|  | Other aggregated species | -4.427 | 1.274 | 8.262 | **0.066** | **0.086** | | | **0.117** |

If a_1_ is greater than zero, then growth of the species increased with SEV. Likewise, if c_1_ is greater than zero, then the competitive effect of the species increases with SEV. Parameters significantly deviated from zero appear in bold font.

The species considered in pair in the model that are followed by similar letters showed non-significantly different growth.
